# 2-Oxoglutarate contributes to the effect of foliar nitrogen on enhancing drought tolerance during flowering and grain yield of soybean

**DOI:** 10.1038/s41598-023-34403-5

**Published:** 2023-05-04

**Authors:** Zhijia Gai, Maoming Zhang, Pengfei Zhang, Jingtao Zhang, Jingqi Liu, Lijun Cai, Xu Yang, Na Zhang, Zhengnan Yan, Lei Liu, Guozhong Feng

**Affiliations:** 1 Jiamusi Branch, Heilongjiang Academy of Agricultural Sciences/Key Laboratory of Breeding and Cultivation of Main Crops in Sanjiang Plain, Jiamusi, 154007 China; 2 College of Resources and Environment, Jilin Agricultural University, Changchun, 130118 China; 3 State Key Laboratory of Black Soils Conservation and Utilization, Northeast Institute of Geography and Agroecology, Chinese Academy of Sciences, Changchun, 130102 China; 4 Department of Agronomy, Northeast Agricultural University, Harbin, 15000 China; 5 College of Horticulture, Qingdao Agricultural University, Qingdao, 266109 China

**Keywords:** Plant stress responses, Plant physiology

## Abstract

Drought severely affects the growth and yield of soybean plants especially during the flowering period. To investigate the effect of 2-oxoglutarate (2OG) in combination with foliar nitrogen (N) at flowering stage on drought resistance and seed yield of soybean under drought stress. This experiment was conducted in 2021 and 2022 on drought-resistant variety (Hefeng 50) and drought-sensitive variety (Hefeng 43) soybean plants treated with foliar N (DS + N) and 2-oxoglutarate (DS + 2OG) at flowering stage under drought stress. The results showed that drought stress at flowering stage significantly increased leaf malonaldehyde (MDA) content and reduced soybean yield per plant. However, superoxide dismutase (SOD), peroxidase (POD) and catalase (CAT) activities were significantly increased by foliar N treatment, and 2-oxoglutarate synergistically with foliar N treatment (DS + N + 2OG) was more beneficial to plant photosynthesis. 2-oxoglutarate significantly enhanced plant N content, glutamine synthetase (GS) and glutamate synthase (GOGAT) activity. Furthermore, 2-oxoglutarate increased the accumulation of proline and soluble sugars under drought stress. Under drought stress, soybean seed yield was increased by DS + N + 2OG treatment by 16.48–17.10% and 14.96–18.84% in 2021 and 2022, respectively. Thus, the combination of foliar N and 2-oxoglutarate better mitigated the adverse effects of drought stress and could better compensate for the yield loss of soybean under drought stress.

## Introduction

Soybean, one of the world's major food crops, is the largest source of vegetable oil and protein^1^. Drought stress limits the growth of soybean, especially when drought occurs during flowering and early pod development, which directly leads to yield reduction^2,3^. Soybean yield is greatly affected by drought stress and the flowering period is characterized by active nutritional and reproductive growth requiring more carbohydrates and nitrogen assimilates^3^. Higher plants respond to drought stress in many strategies, and the gross morphological and physiological responses are well reported^4–6^, such as osmotic adjustment. Proline (Pro) accumulation is a vital osmotic adjustment for plants to acclimate to adverse environmental stresses and has been revealed to occur after drought stress in plants^7,8^. Drought stress could hinder many physiological processes of plants^9^. Plants subjected to drought stress undergo increased accumulation of free radicals and exposure to activated forms of oxygen greatly related to injury to membranes and buildup of lipid peroxides^10^. Plant cells normally are protected against such effects by a complex antioxidant system^11,12^, which is an important strategy protection mechanism for the plant under drought stress. When plants are exposed to abiotic stress, many active oxygen species such as hydrogen peroxide (H_2_O_2_), superoxide (O_2_^−^), hydroxyl radicals, and excessive ammonium (NH_4_^+^) are generated, which impose injury on plants^13,14^. Therefore, it is of great significance for soybean production to take measures to alleviate drought stress and reduce yield loss after drought at the flowering stage.

Nitrogen, as an essential and abundant element of plants, is one of the key environmental factors regulating plant development. Foliar spray of urea can significantly improve crop stress tolerance by protecting photosynthetic apparatus, activating the antioxidant defense system and improving osmoregulation^15,16^. The activities of superoxide dismutase (SOD), peroxidase (POD) and catalase (CAT) were enhanced with nitrogen treatment under drought stress, leading to a reduction in foliar ROS (H_2_O_2_, O_2_^−^ and malonaldehyde, MDA) in maize^15^. Foliar sprays of a mixture of urea and urease inhibitors increased the dry weight, Pro and soluble sugar (SS) content of the crop under drought stress^17^. In addition, foliar sprays of nitrogen-containing regulators increased the photosynthetic rate and SPAD values of rice and were utilized in terms of rice yield in alkalinity-stressed areas^18^. The response of plants to nitrogen fertilization under drought conditions varies depending on the plant species, climate, nitrogen source and fertilization method^19^. The addition of NH_4_^+^ to nutrient solutions has been reported to mitigate the adverse effects of drought on rice growth and development^20^. Although the nitrogen requirement of soybean is not as strong as that of other non-nitrogen fixing crops, due to the strong nitrogen fixation by its root nodules. However, nitrogen application through foliar sprays during flowering has become one of the common means of increasing yield in production^21,22^. Some studies have shown that foliar N sprays can increase soybean yields^23^. However, excessive nitrogen fertilization can cause NH_4_^+^ toxicity in plants^24^. In addition, under drought and salinity conditions, plants can acquire more NH_4_^+^^25^. Even relatively low concentrations of NH_4_^+^ can cause toxicity symptoms, including foliar Necrosis, chloroplast membrane degradation, and disturbed water pressure and osmotic status^26,27^. Plant growth regulators, as a means of crop improvement, are widely applied to agricultural production, which could effectively increase the stress resistance of plants^28^.

2-Oxoglutarate is a key metabolite at the crossroads of C/N metabolism as it is required for ammonia assimilation^29,30^. There is evidence indicating exogenous 2-oxoglutarate has positive effects on nitrogen assimilation and tolerance improvement in crops, such as tobacco^31^ when subjected to abiotic stress. Normally, all forms of nitrogen need to be reduced to NH_4_^+^ before incorporation into the carbon cytoskeleton, through processes that require reduction equivalents and energy^30^. The NH_4_^+^ assimilation is mainly carried out by the action of two enzymes, glutamine synthetase (GS) and glutamate synthase (GOGAT)^32^. The GS-GOGAT cycle requires a carbon skeleton supply of 2-ketoglutarate to operate in order to carry out the linkage step between carbon and nitrogen metabolism and maintain the C-N balance of the plant^30^. Our previous study showed that 2-oxoglutarate significantly increased NH_4_^+^ assimilation and Pro accumulation in soybean seedlings under low temperature stress^33^. However, the mechanism of 2-oxoglutarate effect on foliar N uptake is poorly understood under drought stress and little information is available on the effect of foliar sprays of 2-oxoglutarate on soybean yield.

Therefore, in this study, two soybean varieties with different drought tolerance were selected for combined foliar N and 2-oxoglutarate treatment during flowering under drought stress. In this paper, we firstly report the combined effect of foliar N and 2-oxoglutarate on soybean seed yield under drought stress. We hypothesized that: (1) exogenous 2-oxoglutarate contributes to foliar N uptake and improves drought tolerance in soybean at flowering; and (2) 2-oxoglutarate synergistically compensates for yield loss due to drought stress in soybean at flowering stage.

## Materials and methods

### Plant materials and growth conditions

The pot trial was carried out in the rain shelter at the experimental station of Northeast Agricultural University, China in 2021 and 2022. The diameter and depth for each experimental pot were 30 cm and 45 cm, respectively. All pots were filled with 10 kg of typical black soil taken from a corn field. Two soybean cultivars, Hefeng 50 (drought-resistant variety) and Hefeng 43 (drought-sensitive variety) were used in this experiment. The soybean seeds were obtained from Jiamusi Branch, Heilongjiang Academy of Agricultural Sciences. A completely random design was adopted in the present study. Soil from a corn field was collected at the depth of 20 cm. The properties of the soil were shown in Table 1. The average temperature was 18–33 °C in 2021, 18–34 °C in 2022, and the average day length was 15 h 23 min.

**Table 1:** Soil properties in 2021 and 2022.

Year/parameter	Organic matter (g/kg)	Available nitrogen (mg/kg)	Available phosphorus (mg/kg)	Available potassium (mg/kg)	Available boron (mg/kg)	pH
2021	26.87 ± 4.21	137.34 ± 5.32	36.98 ± 2.94	124.09 ± 6.44	0.23 ± 0.01	6.96 ± 0.12
2022	25.59 ± 3.53	134.21 ± 6.44	36.84 ± 2.41	118.65 ± 6.81	0.21 ± 0.01	6.91 ± 0.09

We state that our experimental research on soybeans comply with the relevant institutional, national, and international guidelines and legislation.

### Experimental design and sampling

Ten seeds of each soybean cultivar were planted by hand in each pot. One seedling was kept per pot. The soil water content under drought stress (DS) was maintained at 50 ± 5% of water-holding capacity and normal soil water content was maintained at 80 ± 5% of water-holding capacity. Foliar N (0.5% Urea) and 2-oxoglutarate (5 mmol/L, v/v) were sprayed for one time on soybean at R2 stage^34^. Five treatments were included in the present experiment: foliar N under drought stress (DS + N), foliar 2-oxoglutarate under drought stress (DS + 2OG), foliar N plus 2-oxoglutarate under drought stress (DS + N + 2OG), foliar distilled water under drought stress (DS), normal soil water content with foliar distilled water (CK). Leaf samples for each treatment were collected after drought stress of 2 days (S1 stage), 4 days (S2 stage) and 6 days (S3 stage). With a weighing method, the weight of each pot was recorded once a day and added to the pot when the relative water content in the soil was lower than the lower limit of the water control treatment^35^.

We state that our experimental research on soybeans comply with the relevant institutional, national, and international guidelines and legislation.

### Analysis of photosynthetic index

Photosynthetic rate (Pn) was determined according to the method of Gai et al.^36^. The photosynthetic rate was measured with the CI-340 portable photosynthesis system. Chlorophyll (Chl) content in the middle leaflet of the trifoliate leaf at the flowering stage (R2) was measured by SPAD-502 Chlorophyll Meter.

### Analysis of membrane damage and antioxidant enzyme activities

Superoxide Dismutase (SOD) activity assay followed the method of Zhang et al.^37^. The method of determining peroxidase (POD) activity and catalase (CAT) activity was described by Liu et al.^38^.

The extent of lipid peroxidation was estimated by determining the content of MDA with the method of Quan et al.^39^.

### Analysis of nitrogen metabolism

Nitrogen content was determined using a Vario MAX-CNS analyzer (Elementar Analyze system GmbH, Hanau, Germany) according to the manufacturer's instructions. The activity of glutamine synthetase (GS) was measured with the method of Lu et al.^40^. One unit of NADH-GDH activity was the reduction amount of 1 µmol of coenzyme (NADH) per min under 30 °C and one unit of GS activity was defined as the amount of enzyme catalyzing the formation of 1 µmol γ-glutamylhydroxamate per min under 37 °C. Glutamate synthase (GOGAT) activity was measured by the reduction of 2-ketoglutarate to glutamate according to the method described by Groat and Vance^41^. The oxidation of 1 µmol min^−1^ of NADH was defined as one unit of GOGAT activity.

### Analysis of Pro metabolism and osmoregulators

The activity of plant pyrroline-5-carboxylate synthase (P5CS) was determined by the method of Fan et al.^42^. One unit of P5CS activity is defined as the reduction of 1 µmol of NADPH per min at 30 ℃. The activity of proline dehydrogenase (ProDH) was determined as described by Fan et al.^42^.

The SS and Pro concentrations were respectively determined with the method of Quan et al.^39^ and Bates et al.^43^.

### Measurement of yield and yield components

The yield components were pod number per plant (PNPP), seed number per plant (SNPP), 100-seed weight (100SW). Seed yield (SY) was weighed and adjusted to 130 g kg^-1^ moisture.

### Statistical analysis

All data were analyzed by using SPSS 22 (SPSS Inc., Chicago, IL, US). A significance test was conducted by variance analysis, and Duncan’s range test was performed to determine the significant differences (*P* < *0.05*) among different treatments for each cultivar. Different lowercase letters above the columns of figures indicate statistically significant differences at *P* < 0.05 (Duncan’s range test). ns stands for not significant.

## Results

### Photosynthetic rate and chlorophyll

**Figure 1:**
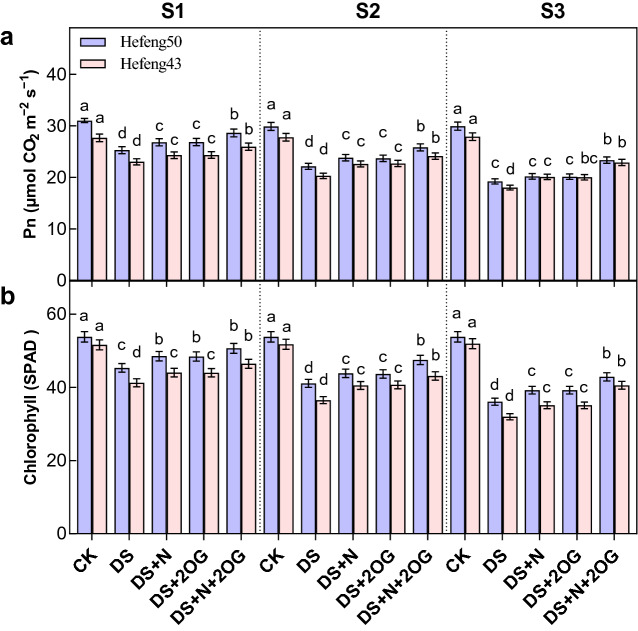
Impacts of foliar nitrogen plus α-ketoglutarate on photosynthetic rate (Pn, **a**) and chlorophyll content (SPAD, **b**) in 2021. CK, normal soil water content with foliar distilled water; DS, foliar distilled water under drought stress; DS + N, foliar nitrogen under drought stress; DS + 2OG, DS + N + 2OG, foliar nitrogen plus α-oxoglutarate under drought stress. S1, day 2 of drought stress; S2, day 4 of drought stress; S3, day 6 of drought stress. Different lowercase letters above the columns of figures indicate statistically significant differences were determined between different treatments for each cultivar at *P* < 0.05 (Duncan’s range test).

Drought significantly reduced photosynthesis and chl content in both varieties of soybean. However, Pn was significantly increased by the foliar N or 2OG treatment under drought stress (Fig. 1). Compared to the DS + N treatment, plant Pn was significantly increased by DS + N + 2OG treatment by 12.74–13.26%, 16.71–18.86% and 21.50–27.03% for both varieties at S1, S2 and S3 stages in 2021, respectively. Similarly, SPAD was significantly increased by foliar N or 2OG treatment under drought stress. Compared to DS + N treatment, the SPAD was significantly increased by DS + N + 2OG treatment by 11.76–12.57%, 15.56–24.16% and 18.82–26.81% in S1, S2 and S3 stages for both cultivars, respectively. Moreover, the trend in 2022 was consistent with that in 2021 (Tables S1 and S2). Under DS + N + 2OG treatment, Pn and SPAD values were slightly higher in Hefeng50 than in Hefeng43. In addition, there was no significant difference in the measured values between DS + N and DS + 2OG treatments.

### Malondialdehyde and antioxidant enzymes

**Figure 2:**
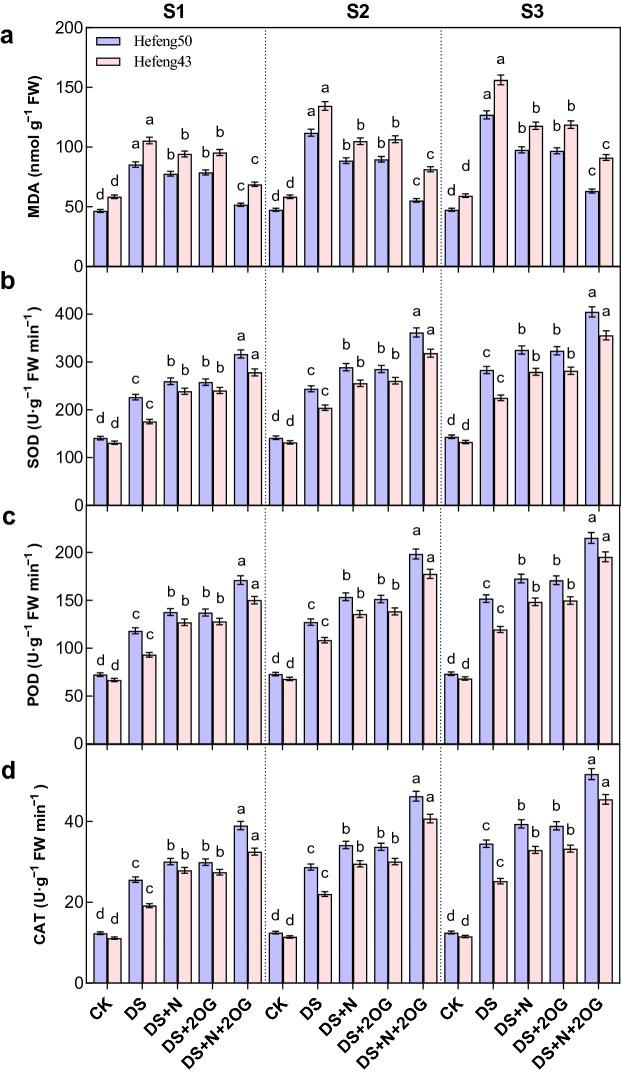
Impacts of foliar nitrogen plus α- ketoglutarate on leaf malondialdehyde (MDA, **a**) content, superoxide dismutase (SOD, **b**) activity, superoxide dismutase (POD, **c**) activity, and catalase (CAT, **d**) activity in 2021. CK, normal soil water content with foliar distilled water; DS, foliar distilled water under drought stress; DS + N, foliar nitrogen under drought stress; DS + 2OG, DS + N + 2OG, foliar nitrogen plus α-oxoglutarate under drought stress. S1, day 2 of drought stress; S2, day 4 of drought stress; S3, day 6 of drought stress. Different lowercase letters above the columns of figures indicate statistically significant differences were determined between different treatments for each cultivar at *P* < 0.05 (Duncan’s range test).

Under drought stress, MDA was significantly reduced by foliar DS + N treatment (Fig. 2). Compared to DS + N treatment, plant MDA levels of both varieties were significantly reduced by N + 2OG treatment by 39.44–35.46%, 50.64–38.84% and 50.32–41.64% at S1, S2 and S3 stages in 2021, respectively. In addition, the MDA level of Hefeng50 was slightly lower than that of Hefeng43 under DS + N + 2OG treatment. In contrast, SOD was significantly increased by foliar DS + N treatment under drought stress. Compared to DS + N treatment, plant SOD was significantly increased by DS + N + 2OG treatment by 39.83–52.51%, 48.23–49.77% and 43.07–58.00% for both varieties at S1, S2 and S3 stages in 2021, respectively. Similarly, POD was significantly increased by foliar DS + N treatment under drought stress. Compared to DS + N treatment, plant POD was significantly increased by DS + N + 2OG treatment by 44.80–53.34%, 55.76–63.92% and 41.78–63.52% in S1, S2 and S3 stages for both cultivars, respectively. Likewise, CAT was significantly increased by the foliar DS + N treatment under drought stress. Compared to DS + N treatment, plant CAT was significantly increased by DS + N + 2OG treatment by 52.35–69.55%, 61.15–84.80% and 49.84–87.58% in S1, S2 and S3 stages for both varieties, respectively. In addition, the trend of the above data was consistent in 2021 with 2022 (Tables S3–S6). The SOD, POD and CAT activities of Hefeng50 were slightly greater than those of Hefeng43 under DS + N + 2OG treatment.

### Nitrogen metabolism

**Figure 3:**
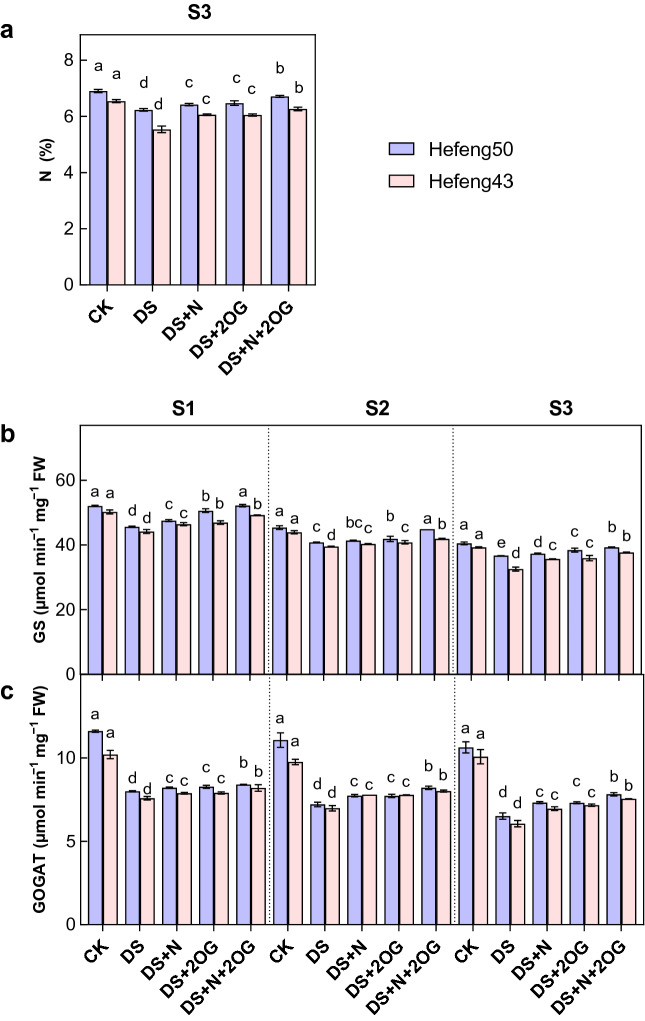
Impacts of foliar nitrogen plus α- ketoglutarate on leaf nitrogen (N, **a**) content, glutamine synthetase (GS, **b**) activity, and glutamate synthase (GOGAT, **c**) activity in 2021. CK, normal soil water content with foliar distilled water; DS, foliar distilled water under drought stress; DS + N, foliar nitrogen under drought stress; DS + 2OG, DS + N + 2OG, foliar nitrogen plus α-oxoglutarate under drought stress. S1, day 2 of drought stress; S2, day 4 of drought stress; S3, day 6 of drought stress. Different lowercase letters above the columns of figures indicate statistically significant differences were determined between different treatments for each cultivar at *P* < 0.05 (Duncan’s range test).

Under drought stress, N content was significantly increased by the foliar DS + N treatment (Fig. 3). Compared to DS + N treatment, plant N content of both varieties were significantly increased by N + 2OG treatment by 7.70–11.69% at S3 stage in 2021. In addition, the N content of Hefeng50 was slightly higher than that of Hefeng43 under DS + N + 2OG treatment. Similarly, GS was significantly increased by the foliar DS + N treatment under drought stress. Compared to DS + N treatment, plant GS activity of both varieties was significantly increased by DS + N + 2OG treatment by 10.28–14.33%, 5.82–9.94% and 7.13–14.72% at S1, S2 and S3 stages in 2021, respectively. Similarly, GOGAT was significantly increased by foliar DS + N treatment under drought stress. Compared to DS + N treatment, plant GOGAT was significantly increased by DS + N + 2OG treatment by 5.02–6.45%, 12.35–13.84% and 19.98–21.67% in S1, S2 and S3 stages for both varieties, respectively. In addition, the N content, GS and GOGAT activities of Hefeng50 were slightly higher than those of Hefeng43 under DS + N + 2OG treatment.

### Proline metabolism and soluble sugars

Pro accumulation during osmotic stress is mainly due to increased synthesis and reduced degradation^44^. In proline synthesis, glutamate is converted to Pro by two successive reductions catalyzed by P5CS and pyrroline-5-carboxylate reductase (P5CR), respectively. The Pro degradation is the reverse process of Pro biosynthesis and catalyzed by Pro dehydrogenase (ProDH) and P5C dehydrogenase (P5CDH). P5CS and ProDH are the key enzymes in proline synthesis and proline degradation, respectively.

**Figure 4:**
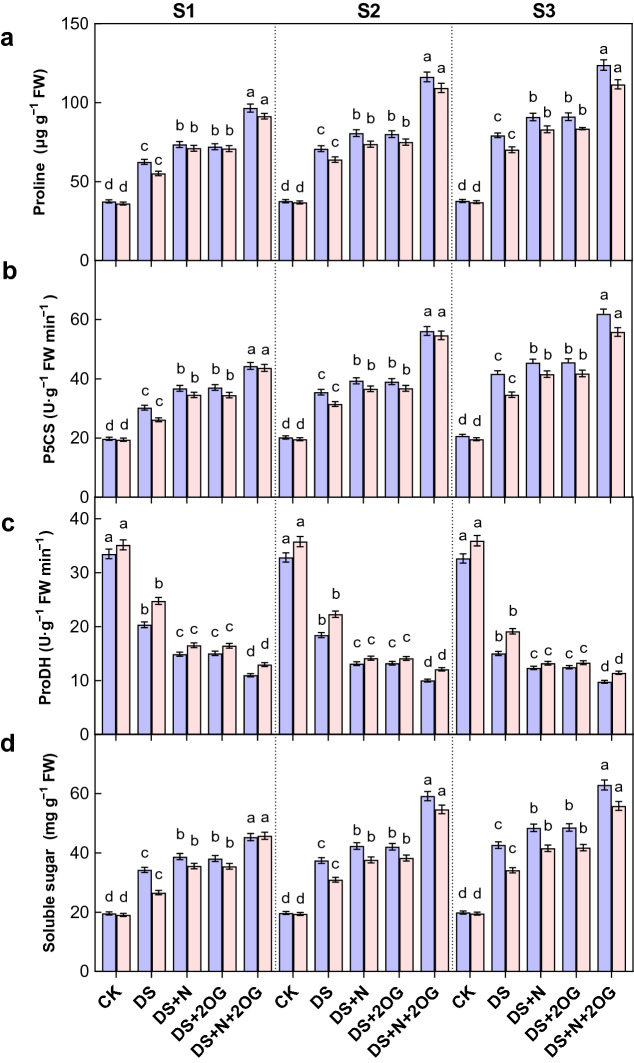
Impacts of foliar nitrogen plus α-ketoglutarate on proline (Pro, **a**) content, pyrroline-5-carboxylate synthase (P5CS, **b**) activity (b), proline dehydrogenase (ProDH, **c**) activity and soluble sugar (SS, **d**) content in 2021. CK, normal soil water content with foliar distilled water; DS, foliar distilled water under drought stress; DS + N, foliar nitrogen under drought stress; DS + 2OG, DS + N + 2OG, foliar nitrogen plus α-oxoglutarate under drought stress. S1, day 2 of drought stress; S2, day 4 of drought stress; S3, day 6 of drought stress. Different lowercase letters above the columns of figures indicate statistically significant differences were determined between different treatments for each cultivar at *P* < 0.05 (Duncan’s range test).

Under drought stress, Pro concentration was significantly increased by the foliar DS + N treatment (Fig. 4). Compared to DS + N treatment, plant proline of both varieties was significantly increased by DS + N + 2OG treatment by 54.57–63.81%, 64.12–70.90% and 56.17–58.99% at S1, S2 and S3 stages in 2021, respectively. Similarly, P5CS activity was significantly increased by the foliar DS + N treatment under drought stress. Compared to DS + N treatment, plant P5CS activity was significantly increased by DS + N + 2OG treatment by 46.45–65.28%, 58.24–73.64% and 48.66–61.23% in S1, S2 and S3 stages for both cultivars, respectively. As shown in Fig. 4, ProDH activity was significantly reduced by foliar DS + N treatment under drought stress. Compared to DS + N treatment, plant ProDH was significantly reduced by DS + N + 2OG treatment by 45.92–47.61%, 42.98–45.58% and 34.91–40.21% in S1, S2 and S3 stages for both cultivars, respectively. In addition, the Pro concentration and P5CS activity of Hefeng50 were slightly higher than that of Hefeng43 under DS + N + 2OG treatment. Compared to DS + N treatment, plant soluble sugar content of both varieties was significantly increased by N + 2OG treatment by 32.27–71.87%, 58.06–76.55% and 47.53–65.19% at S1, S2 and S3 stages in 2021, respectively. Moreover, the trend of the above data was consistent in 2021 with 2022 (Tables S7–S10).

### Yield and yield composition

**Figure 5:**
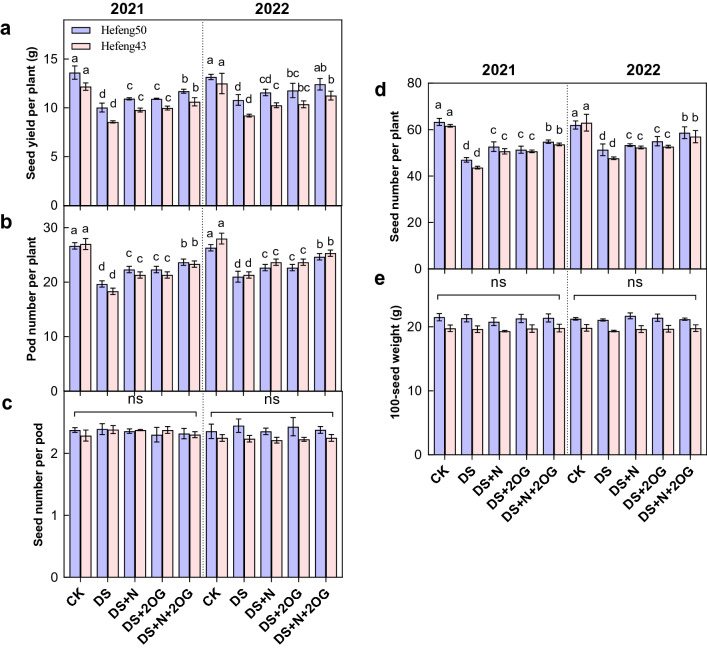
Impacts of foliar nitrogen plus α-ketoglutarate on soybean seed yield per plant (**a**), pod number per plant (**b**), seed number per pod (**c**), seed number per plant (**d**), and 100-seed weight (**e**) in 2021 and 2022. Vertical bars indicate means ± SD (n = 3). CK, normal soil water content with foliar distilled water; DS, foliar distilled water under drought stress; DS + N, foliar nitrogen under drought stress; DS + 2OG, DS + N + 2OG, foliar nitrogen plus α-oxoglutarate under drought stress. S1, day 2 of drought stress; S2, day 4 of drought stress; S3, day 6 of drought stress. Different lowercase letters above the columns of figures indicate statistically significant differences were determined between different treatments for each cultivar at *P* < 0.05 (Duncan’s range test). ns, no significant.

Drought significantly reduced seed yield and yield components of both varieties of soybean. However, seed yield was significantly increased by the foliar DS + N or 2OG treatment under drought stress (Fig. 5). In comparison to DS + N treatment, seed yield per plant (SYPP) was significantly increased by N + 2OG treatment by 16.48–17.10% in 2021 for both varieties, respectively. In 2022, the seed yield per plant of both varieties was significantly increased by N + 2OG treatment by 14.96–18.84%, respectively. Similarly, pod number per plant (PNPP) was significantly increased by the foliar DS + N treatment under drought stress. Compared to DS + N treatment, the pod number per plant was significantly increased by DS + N + 2OG treatment by 20.34–27.78% for both varieties in 2021, respectively. In 2022, the pod number per plant of both varieties was significantly increased by N + 2OG treatment by 17.46–19.05%, respectively. Similarly, seed number per plant was significantly increased by foliar DS + N and DS + 2OG treatments under drought stress. In addition, Seed number per pod (SNPP) and 100–seed weight were not significantly different under each treatment. In addition, there was no significant difference in the measured values between DS + N and DS + 2OG treatments.

### Multivariate statistical analysis

**Figure 6:**
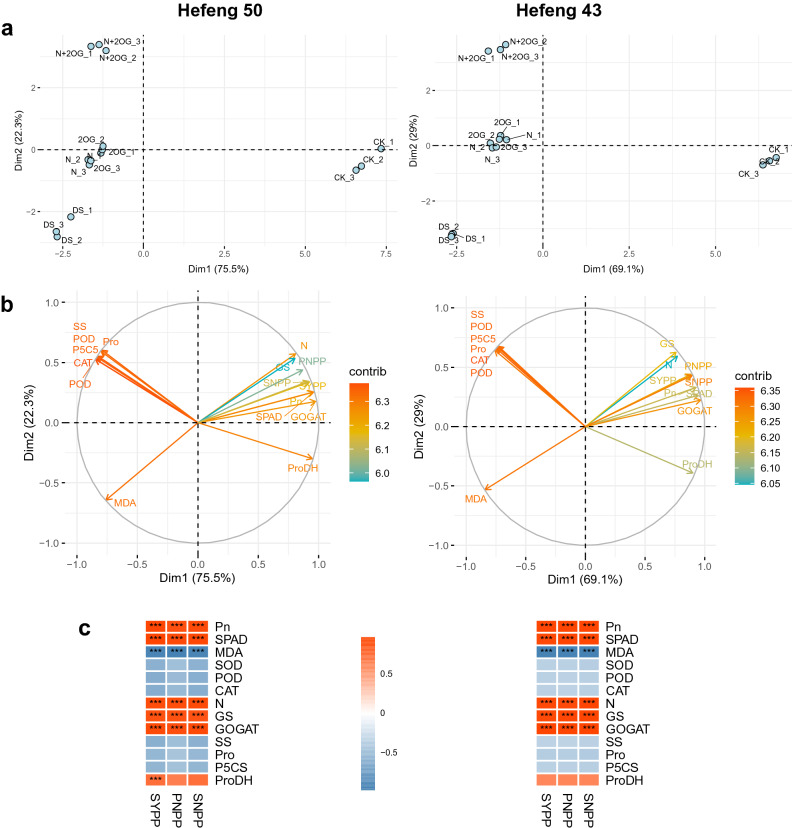
Principal component analysis (PCA, **a**), biplots analysis (**b**) and correlation (**c**) among main indicators of the two genotypes of plants under drought stress. The red dotted line represents the average value of the variables in the graph. Pn, photosynthetic rate; MDA, malonaldehyde; SOD, superoxide dismutase; POD, peroxidase; CAT, catalase; N, nitrogen; GS, glutamine synthetase; GOGAT, glutamate synthase; SS, soluble sugar; Pro, proline;P5CS, pyrroline-5-carboxylate synthase; ProDH, proline dehydrogenase; SYPP, seed yield per plant; PNPP, pod number per plant; SNPP, seed number per plant.

Principal component analysis (PCA) was performed on important data to explain the regulation mode of N and 2OG treatment on drought stress (Fig. 6). Physiological and yield data of each genotype were displayed in the biplots plots (Fig. 6b). The treatment and genotype were well separated in the plots. The directional movement index (Dim) 1 and Dim2 accounted for 69.1–75.5% and 29.0–22.3% of the variation respectively. The Dim1 clearly distinguished the effects of CK and other treatments, while Dim2 determined the parameter differences between DS and DS + 2OG treatments. The contributions of CAT and POD were ranked in the top 2 in the contribution of biplots plot. In both varieties, Pn, SPAD, N, GS and GOGAT were correlated with yield components (SYPP, PNPP and SNPP) were highly significantly positively correlated (*P* < 0.001). MDA was highly significantly negatively correlated with all yield constitutive factors. In addition, ProDH was highly significantly and positively correlated with SYPP in Hefeng50.

## Discussion

### Impacts of foliar N plus 2-oxoglutarate on Pn and Chl during flowering under drought stress

Photosynthesis is closely related to plant growth and seed yield, and Pn and Chl are two key parameters of photosynthesis^45^. Drought adversely affects total chl content, which may be due to inhibition of chlorophyll biosynthesis, ROS-mediated degradation and suppression of the activity of nitrogen metabolism^46^. The data shown in this study show that foliar N and 2-oxoglutarate significantly promoted photosynthesis during the flowering stage of soybean under drought stress (Fig. 1). When several seedlings were subjected to low salinity, nitrogen fertilization significantly increased Pn and N concentrations in tree needles and roots, thus improving seedling height and total biomass of both species^47^. In the present study, 2-oxoglutarate increased photosynthesis in soybean under drought stress under foliar N treatment. Alamri^45^ reported a positive effect of 2-oxoglutarate triggering on photosynthesis and chl of tomato seedlings under heavy metal stress. In addition, organic acids such as 2-oxoglutarate act in plants to provide redox homeostasis, support ionic gradients across membranes and acidification of the extracellular medium, contributing to metabolic homeostasis^48^. Therefore, these results suggest that 2-oxoglutarate can protect chlorophyll and photosynthesis in soybean leaves in response to drought stress.

### Impacts of foliar N plus 2-oxoglutarate on ROS metabolism during flowering under drought stress

Drought exacerbates the accumulation of reactive oxygen species (ROS) in plant photosynthesis and respiration. Therefore, avoiding oxidative stress of these compounds is essential for normal cellular function^49^. MDA is considered a key indicator of the degree of drought stress^50,51^. In the present study, the MDA content of both soybean cultivars under drought stress gradually increased with the duration of stress, while both foliar N and 2-oxoglutarate significantly reduced the MDA content of both soybean cultivars under drought stress (Fig. 2). In cyanobacteria, crosstalk between ROS homeostasis and nitrogen metabolism proceeds through a mechanism independent of known redox regulators. Robles-Rengel et al. suggested that ROS could sense global nitrogen regulation by reducing intracellular concentrations of 2-oxoglutarate^49^. The results showed that higher N nutrition promotes drought tolerance in wheat by maintaining higher photosynthetic activity and antioxidant defense system during the nutritional growth period^52^. 2-oxoglutarate could better reduce the accumulation of MDA under foliar N treatment under drought stress. It has been shown that 2-oxoglutarate triggering can significantly reduce As-induced ROS production in tomato seedlings as well as improved membrane stability through antioxidant enzymes^45^. SOD, CAT and POD are important antioxidant enzymes for detoxification of reactive oxygen species^11–13^. Here, 2-oxoglutarate significantly increased the activities of SOD, CAT and POD in plants at all stages of foliar N treatment under drought stress. Beside the role of 2-oxoglutarate in biosynthetic processes, its role in regulating antioxidant enzyme activities and cellular redox status has also been reported^53,54^. 2-oxoglutarate can also reduce the accumulation of ROS and its associated damage to lipids, proteins and membranes by regulating antioxidant enzymes (GST), thus protecting arsenic (As) toxicity in of tomato roots^45^. Thus, exogenous 2-oxoglutarate in concert with foliar N protected ROS homeostasis in flowering soybean plants under drought stress.

### Impacts of foliar N plus 2-oxoglutarate on N metabolism during flowering under drought stress

The activities of key enzymes involved in plant nitrogen metabolism reflect the overall level of nitrogen assimilation and nitrogen metabolism^55^. In the present study, soybean leaf GS and GOGAT activities were significantly reduced under drought stress (Fig. 3), which may be due to a chain reaction caused by substrate deficiency. Under abiotic conditions, exogenous nitrogen has been shown to enhance leaf nitrogen metabolism and the activities of key enzymes involved in nitrogen metabolism, while reducing the rate of leaf aging by improving soybean stress tolerance through regulation of nitrogen metabolism^52^. In this study, foliar N and 2-oxoglutarate significantly increased the N content of two soybean cultivars under drought stress. Interestingly, foliar 2-oxoglutarate increased the N content of soybean under foliar N treatment under drought stress. Proper regulation of carbon and N assimilation is a key factor in crop productivity, and 2-oxoglutarate regulates the catabolism and anabolism of TCA products and substrates by regulating the production of amino acids, NAD+/NADH, and ATP^56,57^. It has been shown that N addition promotes N nutrition in plants under salt stress^47^. It was also found that the addition of 2-oxoglutarate to NO_3_^-^ containing nutrients enhanced the expression of NiR, GDH and antioxidant gene SOD in potato roots^58^. Here, foliar N and 2-oxoglutarate significantly increased GS and GOGAT activities in both soybean cultivars under drought stress. Exogenous 2-oxoglutarate could contribute to maintain a relatively stable N assimilation capacity under drought conditions by upregulating key enzyme activities^59^. Therefore, these findings contribute to a better understanding of how exogenous 2-oxoglutarate regulates N uptake and C/N under drought stress, which may help to improve N use efficiency in soybean leaves.

### Impacts of foliar N plus 2-oxoglutarate on proline metabolism during flowering under drought stress

Proline and soluble sugars are important osmoregulatory products, and their accumulation helps maintain high intracellular water potential and osmotic pressure, thus effectively protecting cell membrane stability^59,60^. In the present study, drought stress resulted in a significant increase in proline and soluble protein content in soybean plants, which was further promoted by exogenous application of foliar N and 2-oxoglutarate during drought stress (Fig. 4). These results suggest that 2-oxoglutarate synergistically with foliar N contributes to the maintenance of normal cellular osmotic pressure and thus reduces water loss^33^. It is known that P5CS is a key enzyme for proline synthesis and ProDH is a key enzyme for proline degradation^61^. Evidence for the role of nitrogen in mitigating the adverse effects of drought stress by increasing proline accumulation and soluble sugar content^62,63^. Data showed that 2-oxoglutarate significantly promoted the accumulation of proline and soluble sugar content under foliar N treatment. 2-oxoglutarate has been shown to positively affect the energy status of cells by regulating the synthesis of amino acids such as glutamate, glutamine and proline^45^. N is an important regulator of photosynthetic C flow in plants, and plant development requires an adequate balance between N and C metabolism. However, insufficient supply of C can alter this balance, and 2-oxoglutarate is directly involved in the N assimilation process^64^. The current study showed that 2-oxoglutarate significantly increased P5CS activity and decreased ProDH activity under leaf foliar N treatment in soybean subjected to drought stress at flowering. Under environmental stress conditions, such as low temperature, salt and drought stress, proline accumulation was significant due to increased proline synthesis and decreased proline degradation^65^. In the present study, it was observed that drought stress led to an increase in P5CS activity. However, in the present study, ProDH activity was significantly reduced after exposure to drought stress. Both leaf proline content and P5CS activity under drought stress gradually increased with the duration of stress, while ProDH activity gradually decreased, which favored the accumulation of proline.

### Impacts of foliar N plus 2-oxoglutarate on seed yield and yield components of soybean under drought stress

Flowering drought stress significantly reduced soybean yield per plant and yield was inversely proportional to drought duration^66^. The data showed (Fig. 5) that drought stress at flowering significantly reduced seed yield per plant, the number of pods per plant, and the number of seeds per plant in both soybean varieties. Yield components are morphological traits whose formation is essential for yield. Leaf nitrogen and 2-oxoglutarate significantly increased the number of pods per plant and yield per plant in drought-stressed soybean. However, leaf nitrogen and 2-oxoglutarate did not significantly affect the number of seeds per pod and 100-seed weight. The number of pods per plant is a component of seed yield and is easily affected by changes in environmental conditions^67^. Seed yield is determined by seed number and seed quality; however, most environmentally induced yield differences are due to seed number^68^. In the current study, the differences in seed number were mainly due to the number of pods per plant, as there was no significant effect of different treatments on the number of seeds per pod when exposed to drought stress at anthesis. Herbert^69^ stated that the number of seeds per pod was a minor factor in determining seed yield in soybean. The little variation in 100-seed weight and number of seeds per pod under different treatments indicated that 100-seed weight and number of seeds per pod were strongly influenced by internal genetic mechanisms. The combination of foliar N and 2-oxoglutarate, especially foliar N and 2-oxoglutarate, partially compensated for the loss of soybean yield per plant. Under severe drought stress, plants under low N exhibited accelerated seed filling rates and shortened seed filling duration after anthesis and reduced grain yield^52^. Plants under high N exhibited delayed senescence and lower grain yield reductions. High nitrate doses greatly increased amino acid production, which resulted in lower 2-oxoglutarate concentrations^64^. In the present study, 2-oxoglutarate synergistically with foliar N increased soybean yield per plant, probably because of better maintenance of C/N balance and improved nitrogen utilization by 2-oxoglutarate. Therefore, 2-oxoglutarate is beneficial in synergizing with foliar N to improve seed yield when soybean is subjected to drought stress at flowering. It has been reported that cold-resistant soybean variety could better adapt to cold-sensitive variety when subjected to cold stress^52^. Zhang et al.^17^ found that foliar nitrogen application was beneficial to nitrogen metabolism and plant growth in two maize cultivars under and drought-resistant maize variety could better adapt to short-term moderate stress than drought-sensitive variety. Similarly, the photosynthesis, proline content, antioxidant enzyme activity and N metabolism were significantly higher for Hefeng50 than Hefeng43 in the present study and thus it was observed that there was higher seed yield per plant for Hefeng50 than Hefeng43 when exposed to drought stress, suggesting that drought-resistant variety Hefeng50 could better acclimate to drought stress compared to drought-sensitive variety Hefeng43.

2-Oxoglutarate (2OG) is a key metabolite at the crossroads of C/N metabolism as it is required for ammonia assimilation^29,30^. The evidence suggests that exogenous 2-oxoglutarate has positive effects on nitrogen assimilation and tolerance improvement in crops, such as tobacco when exposed to abiotic stress^31^. Similarly, foliar sprays of nitrogen-containing regulators increased the photosynthetic rate and SPAD values of rice and were utilized in terms of rice yield in alkalinity-stressed areas^18^. Based on the previous reports, we infer that foliar application of exogenous 2OG indirectly enhanced the nitrogen assimilation, while foliar application of N directly was beneficial to nitrogen metabolism when exposed to drought stress. Therefore, our results showed that DS + N and DS + 2OG had a similar effect on most indicators.

## Conclusions

**Figure 7:**
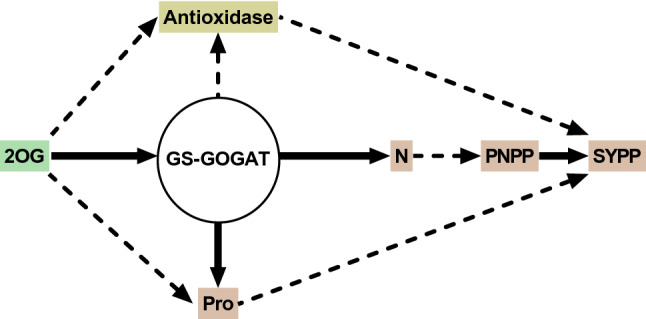
A schematic diagram of alpha-ketoglutaric acid synergistic with foliar N to improve drought tolerance in soybean during flowering stage. The dashed lines represent possible indirect effects and the solid lines represent direct effects. N, nitrogen; GS, glutamine synthetase; GOGAT, glutamate synthase; SS, soluble sugar; Pro, proline; SYPP, seed yield per plant; PNPP, pod number per plant.

This study adds new information that 2-oxoglutarate synergistically with foliar N improved drought tolerance and increased yield per plant in soybean under drought stress at flowering stage. Co-regulation of foliar N by 2-oxoglutarate through increased N uptake, N metabolism, and proline accumulation also increased POD, SOD, CAT activity, photosynthesis, and reduced MDA content under drought stress (Fig. 7). Importantly, 2-oxoglutarate synergistically with foliar N significantly increased soybean pod number and seed yield when soybean was exposed to drought stress at anthesis. The combination of foliar N with 2-oxoglutarate salt was advantageous in mitigating the adverse effects of drought stress and better compensated for the loss of soybean seed yield. Therefore, foliar N spray of synergy with 2-oxoglutarate is a necessary and feasible way to cope with drought stress. In the present study, drought-resistant variety Hefeng50 could better acclimate to drought stress than drought-sensitive variety Hefeng43. In addition, oxoglutarate contributes to the effect of foliar nitrogen on enhancing drought tolerance during flowering and grain yield of soybean.

## Supplementary Information

Supplementary Tables.

## Data Availability

The data presented in this study are available on request from the corresponding author.
