# Supplementary material for: 2-Oxoglutarate contributes to the effect of foliar nitrogen on enhancing drought tolerance during flowering and grain yield of soybean
Source: Sci Rep. 2023 May 4;13:7274. doi: 10.1038/s41598-023-34403-5 (PMC10160060; doi:10.1038/s41598-023-34403-5)
Supplement: Supplementary file 1 — Supplementary Tables. [file 41598_2023_34403_MOESM1_ESM.docx]

**Supplementary data**

Table S1. Impacts of foliar nitrogen plus α-ketoglutarate on photosynthetic rate (Pn, µmol CO_2_ m^-2^·s^-1^) in 2022

| Treatment | S1stage | | S2 stage | | S3 stage | |
| --- | --- | --- | --- | --- | --- | --- |
|  | Hefeng50 | Hefeng 43 | Hefeng50 | Hefeng 43 | Hefeng50 | Hefeng 43 |
| CK | 32.04±0.847a | 31.63±0.837a | 32.06±0.847a | 31.76±0.841a | 32.31±0.856a | 31.88±0.847a |
| DS | 19.23±0.508d | 17.96±0.476d | 17.92±0.476d | 15.65±0.414d | 15.41±0.406d | 13.96±0.370d |
| DS+N | 23.45±0.618c | 22.43±0.592c | 22.03±0.582c | 20.07±0.529c | 20.84±0.556c | 19.06±0.503c |
| DS+2OG | 24.32±0.644c | 22.05±0.582c | 21.95±0.582c | 20.49±0.539c | 20.02±0.529c | 19.45±0.512c |
| DS+N+2OG | 27.38±0.724b | 24.88±0.661b | 24.14±0.635b | 23.13±0.609b | 22.99±0.609b | 20.41±0.539b |

Values within a column followed different letters are significantly different at 0.05 level. CK, normal soil water content with foliar distilled water; DS, foliar distilled water under drought stress; DS+N, foliar nitrogen under drought stress; DS+2OG, DS+N+2OG, foliar nitrogen plus α-oxoglutarate under drought stress. S1, day 2 of drought stress; S2, day 4 of drought stress; S3, day 6 of drought stress.

Table S2. Impacts of foliar nitrogen plus α-ketoglutarate on chlorophyll content (SPAD) in 2022

| Treatment | S1 stage | | S2 stage | | S3 stage | |
| --- | --- | --- | --- | --- | --- | --- |
|  | Hefeng50 | Hefeng 43 | Hefeng50 | Hefeng 43 | Hefeng50 | Hefeng 43 |
| CK | 52.45±1.385a | 50.55±1.340a | 52.63±1.393a | 50.79±1.344a | 52.74±1.393a | 50.83±1.344a |
| DS | 39.88±1.058d | 37.29±0.988d | 36.15±0.952d | 32.11±0.847d | 32.75±0.864d | 28.13±0.741d |
| DS+N | 43.66±1.155c | 39.61±1.049c | 39.13±1.032c | 36.46±0.962c | 35.27±0.935c | 31.68±0.837c |
| DS+2OG | 43.97±1.164c | 39.76±1.053c | 38.36±1.015c | 36.32±0.962c | 35.04±0.926c | 31.43±0.830c |
| DS+N+2OG | 49.13±1.296b | 46.04±1.217b | 47.05±1.244b | 41.82±1.106b | 44.58±1.181b | 35.02±0.926b |

Values within a column followed different letters are significantly different at 0.05 level. CK, normal soil water content with foliar distilled water; DS, foliar distilled water under drought stress; DS+N, foliar nitrogen under drought stress; DS+2OG, DS+N+2OG, foliar nitrogen plus α-oxoglutarate under drought stress. S1, day 2 of drought stress; S2, day 4 of drought stress; S3, day 6 of drought stress.

Table S3. Impacts of foliar nitrogen plus α- ketoglutarate on leaf MDA content (nmol·g^-1^ FW) in 2022

| Treatment | S1 stage | | S2 stage | | S3 stage | |
| --- | --- | --- | --- | --- | --- | --- |
|  | Hefeng50 | Hefeng 43 | Hefeng50 | Hefeng 43 | Hefeng50 | Hefeng 43 |
| CK | 39.25±1.038d | 51.83±1.370d | 39.04±1.032d | 51.02±1.349d | 39.16±1.032d | 51.18±1.355d |
| DS | 81.54±2.160a | 95.97±2.540a | 98.11±2.593a | 117.78±3.116a | 110.65±2.927a | 136.79±3.619a |
| DS+N | 72.84±1.931b | 86.52±2.292b | 81.48±2.153b | 94.36±2.496b | 90.16±2.381b | 105.91±2.804b |
| DS+2OG | 71.22±1.884b | 85.05±2.249b | 80.81±2.137b | 82.76±2.190b | 89.73±2.372b | 103.39±2.735b |
| DS+N+2OG | 53.58±1.419c | 60.21±1.593c | 57.54±1.525c | 70.57±1.869c | 61.21±1.620c | 72.24±1.911c |

Values within a column followed different letters are significantly different at 0.05 level. CK, normal soil water content with foliar distilled water; DS, foliar distilled water under drought stress; DS+N, foliar nitrogen under drought stress; DS+2OG, DS+N+2OG, foliar nitrogen plus α-oxoglutarate under drought stress. S1, day 2 of drought stress; S2, day 4 of drought stress; S3, day 6 of drought stress.

Table S4. Impacts of foliar nitrogen plus α- ketoglutarate on leaf SOD activity (U·g^-1^ FW min^-1^) in 2022

| Treatment | S1 stage | | S2 stage | | S3 stage | |
| --- | --- | --- | --- | --- | --- | --- |
|  | Hefeng50 | Hefeng 43 | Hefeng50 | Hefeng 43 | Hefeng50 | Hefeng 43 |
| CK | 101.53±2.689d | 91.78±2.428d | 102.01±2.699d | 92.29±2.444d | 102.32±2.708d | 92.72±2.451d |
| DS | 167.24±4.424c | 137.68±3.642c | 175.96±4.657c | 146.22±3.868c | 191.56±5.070c | 152.53±4.039c |
| DS+N | 201.87±5.344b | 187.48±4.957b | 222.33±5.883b | 203.30±5.380b | 252.31±6.677b | 221.71±5.864b |
| DS+2OG | 204.32±5.407b | 185.87±4.921b | 228.68±6.049b | 206.71±5.467b | 259.53±6.870b | 220.24±5.826b |
| DS+N+2OG | 253.37±6.703a | 222.59±5.891a | 289.25±7.652a | 254.75±6.737a | 324.04±8.572a | 284.61±7.531a |

Values within a column followed different letters are significantly different at 0.05 level. CK, normal soil water content with foliar distilled water; DS, foliar distilled water under drought stress; DS+N, foliar nitrogen under drought stress; DS+2OG, DS+N+2OG, foliar nitrogen plus α-oxoglutarate under drought stress. S1, day 2 of drought stress; S2, day 4 of drought stress; S3, day 6 of drought stress.

Table S5. Impacts of foliar nitrogen plus α- ketoglutarate on leaf POD activity (U·g^-1^ FW min^-1^) in 2022

| Treatment | S1 stage | | S2 stage | | S3 stage | |
| --- | --- | --- | --- | --- | --- | --- |
|  | Hefeng50 | Hefeng 43 | Hefeng50 | Hefeng 43 | Hefeng50 | Hefeng 43 |
| CK | 61.56±1.631d | 56.83±1.502d | 62.04±1.640d | 57.98±1.535d | 62.16±1.640d | 58.01±1.535d |
| DS | 110.92±2.937c | 86.65±2.292c | 118.44±3.131c | 100.81±2.667c | 142.16±3.757c | 112.17±2.969c |
| DS+N | 125.32±3.317b | 115.62±3.060b | 139.82±3.698b | 123.55±3.271b | 157.14±4.154b | 135.05±3.572b |
| DS+2OG | 124.35±3.290b | 113.76±3.010b | 138.98±3.678b | 122.43±3.237b | 156.43±4.137b | 134.37±3.555b |
| DS+N+2OG | 162.02±4.286a | 145.66±3.853a | 191.42±5.063a | 172.35±4.560a | 218.73±5.785a | 189.62±5.017a |

Values within a column followed different letters are significantly different at 0.05 level. CK, normal soil water content with foliar distilled water; DS, foliar distilled water under drought stress; DS+N, foliar nitrogen under drought stress; DS+2OG, DS+N+2OG, foliar nitrogen plus α-oxoglutarate under drought stress. S1, day 2 of drought stress; S2, day 4 of drought stress; S3, day 6 of drought stress.

Table S6. Impacts of foliar nitrogen plus α- ketoglutarate on leaf CAT activity (U·g^-1^ FW min^-1^) in 2022

| Treatment | S1 stage | | S2 stage | | S3 stage | |
| --- | --- | --- | --- | --- | --- | --- |
|  | Hefeng50 | Hefeng 43 | Hefeng50 | Hefeng 43 | Hefeng50 | Hefeng 43 |
| CK | 14.05±0.370d | 12.69±0.335d | 14.24±0.376d | 12.87±0.344d | 14.28±0.380d | 13.01±0.344d |
| DS | 26.66±0.705c | 20.13±0.529c | 30.12±0.794c | 23.13±0.609c | 36.24±0.958c | 26.47±0.697c |
| DS+N | 36.09±0.952b | 33.57±0.890b | 40.94±1.085b | 35.42±0.935b | 55.26±1.465b | 49.52±1.313b |
| DS+2OG | 36.87±0.979b | 33.76±0.894b | 40.85±1.085b | 36.78±0.973b | 56.88±1.508b | 48.81±1.291b |
| DS+N+2OG | 50.46±1.332a | 46.65±1.234a | 57.31±1.518a | 51.16±1.349a | 66.19±1.752a | 56.56±1.499a |

Values within a column followed different letters are significantly different at 0.05 level. CK, normal soil water content with foliar distilled water; DS, foliar distilled water under drought stress; DS+N, foliar nitrogen under drought stress; DS+2OG, DS+N+2OG, foliar nitrogen plus α-oxoglutarate under drought stress. S1, day 2 of drought stress; S2, day 4 of drought stress; S3, day 6 of drought stress.

Table S7. Impacts of foliar nitrogen plus α- ketoglutarate on proline content (µg g^-1^ FW) in 2022

| Treatment | S1 stage | | S2 stage | | S3 stage | |
| --- | --- | --- | --- | --- | --- | --- |
|  | Hefeng50 | Hefeng 43 | Hefeng50 | Hefeng 43 | Hefeng50 | Hefeng 43 |
| CK | 42.02±1.173d | 40.49±1.123d | 42.21±1.183d | 41.27±1.153d | 42.31±1.183d | 41.48±1.153d |
| DS | 59.86±1.709c | 53.91±1.530c | 63.55±1.818c | 57.62±1.639c | 71.76±2.057c | 63.53±1.818c |
| DS+N | 70.86±2.037b | 69.91±2.007b | 78.55±2.267b | 76.62±2.207b | 88.76±2.566b | 82.53±2.386b |
| DS+2OG | 70.72±2.027b | 69.55±1.998b | 78.52±2.267b | 76.04±2.187b | 89.35±2.586b | 81.89±2.366b |
| DS+N+2OG | 92.78±2.686a | 87.76±2.536a | 111.68±3.255a | 104.97±3.055a | 118.91±3.474a | 107.16±3.115a |

Values within a column followed different letters are significantly different at 0.05 level. CK, normal soil water content with foliar distilled water; DS, foliar distilled water under drought stress; DS+N, foliar nitrogen under drought stress; DS+2OG, DS+N+2OG, foliar nitrogen plus α-oxoglutarate under drought stress. S1, day 2 of drought stress; S2, day 4 of drought stress; S3, day 6 of drought stress.

Table S8. Impacts of foliar nitrogen plus α- ketoglutarate on leaf P5CS activity (U·g^-1^ FW min^-1^) in 2022

| Treatment | S1 stage | | S2 stage | | S3 stage | |
| --- | --- | --- | --- | --- | --- | --- |
|  | Hefeng50 | Hefeng 43 | Hefeng50 | Hefeng 43 | Hefeng50 | Hefeng 43 |
| CK | 29.01±0.767d | 26.94±0.714d | 29.14±0.767d | 27.11±0.714d | 29.21±0.773d | 27.25±0.720d |
| DS | 40.91±1.085c | 35.94±0.952c | 44.37±1.174c | 38.41±1.015c | 48.84±1.296c | 42.35±1.121c |
| DS+N | 48.01±1.270b | 43.61±1.155b | 53.78±1.423b | 50.75±1.340b | 59.17±1.567b | 56.02±1.482b |
| DS+2OG | 49.74±1.313b | 44.97±1.191b | 54.15±1.429b | 51.44±1.359b | 61.62±1.631b | 56.48±1.491b |
| DS+N+2OG | 62.27±1.650a | 58.97±1.561a | 74.95±1.984a | 70.45±1.861a | 79.81±2.111a | 71.92±1.905a |

Values within a column followed different letters are significantly different at 0.05 level. CK, normal soil water content with foliar distilled water; DS, foliar distilled water under drought stress; DS+N, foliar nitrogen under drought stress; DS+2OG, DS+N+2OG, foliar nitrogen plus α-oxoglutarate under drought stress. S1, day 2 of drought stress; S2, day 4 of drought stress; S3, day 6 of drought stress.

Table S9. Impacts of foliar nitrogen plus α- ketoglutarate on leaf ProDH activity (U·g^-1^ FWmin^-1^) in 2022

| Treatment | S1 stage | | S2 stage | | S3 stage | |
| --- | --- | --- | --- | --- | --- | --- |
|  | Hefeng50 | Hefeng 43 | Hefeng50 | Hefeng 43 | Hefeng50 | Hefeng 43 |
| CK | 35.35±0.935a | 36.91±0.979a | 35.56±0.943a | 37.15±0.979a | 35.68±0.943a | 37.22±0.985a |
| DS | 22.34±0.592b | 27.26±0.724b | 19.33±0.512b | 24.53±0.652b | 16.56±0.440b | 21.07±0.556b |
| DS+N | 13.57±0.361c | 15.07±0.397c | 11.98±0.317c | 13.89±0.370c | 11.24±0.297c | 12.08±0.317c |
| DS+2OG | 13.36±0.353c | 15.39±0.406c | 12.37±0.327c | 13.64±0.361c | 11.74±0.308c | 12.31±0.327c |
| DS+N+2OG | 10.65±0.282d | 12.59±0.335d | 9.72±0.255d | 11.55±0.308d | 9.53±0.255d | 11.14±0.291d |

Values within a column followed different letters are significantly different at 0.05 level. CK, normal soil water content with foliar distilled water; DS, foliar distilled water under drought stress; DS+N, foliar nitrogen under drought stress; DS+2OG, DS+N+2OG, foliar nitrogen plus α-oxoglutarate under drought stress. S1, day 2 of drought stress; S2, day 4 of drought stress; S3, day 6 of drought stress.

Table S10. Impacts of foliar nitrogen plus α- ketoglutarate on soluble sugar content (mg g^-1^ FW) in 2022

| Treatment | S1 stage | | S2 stage | | S3 stage | |
| --- | --- | --- | --- | --- | --- | --- |
|  | Hefeng50 | Hefeng 43 | Hefeng50 | Hefeng 43 | Hefeng50 | Hefeng 43 |
| CK | 27.56±0.731d | 26.56±0.705d | 27.76±0.735d | 26.61±0.705d | 27.96±0.741d | 26.97±0.714d |
| DS | 33.93±0.900c | 30.96±0.820c | 36.78±0.973c | 33.81±0.894c | 37.88±1.005c | 34.75±0.917c |
| DS+N | 41.27±1.094b | 37.81±1.000b | 46.37±1.226b | 42.52±1.128b | 51.46±1.359b | 48.71±1.287b |
| DS+2OG | 42.29±1.121b | 38.64±1.022b | 46.62±1.234b | 43.24±1.143b | 52.64±1.393b | 48.49±1.279b |
| DS+N+2OG | 49.26±1.306a | 45.83±1.211a | 59.48±1.570a | 50.28±1.332a | 68.76±1.820a | 58.27±1.544a |

Values within a column followed different letters are significantly different at 0.05 level. CK, normal soil water content with foliar distilled water; DS, foliar distilled water under drought stress; DS+N, foliar nitrogen under drought stress; DS+2OG, DS+N+2OG, foliar nitrogen plus α-oxoglutarate under drought stress. S1, day 2 of drought stress; S2, day 4 of drought stress; S3, day 6 of drought stress.
